# Implementation of a targeted treatment strategy for the sustainable control of *Ascaridia galli* infections in laying hens

**DOI:** 10.1002/vro2.37

**Published:** 2022-05-30

**Authors:** Behdad Tarbiat, Désirée Seger Jansson, Johan Höglund

**Affiliations:** ^1^ Department of Biomedical Sciences and Veterinary Public Health, Section for Parasitology Swedish University of Agricultural Sciences (SLU) Uppsala Sweden; ^2^ Department of Clinical Sciences Swedish University of Agricultural Sciences (SLU) Uppsala Sweden; ^3^ Department of Animal Health and Antimicrobial Strategies National Veterinary Institute (SVA) Uppsala Sweden

## Abstract

**Background:**

*Ascaridia galli* is a widespread problem in cage‐free egg production. Sustainable control of nematode infections is a key component in this sector. This study investigates the effect of a treatment strategy against *A. galli*, aiming to propose a guideline for anthelmintic use on commercial poultry farms.

**Methods:**

A total of eight flocks of laying hens (a–h) from five commercial poultry farms were included in this study. Faecal samples were collected on a biweekly basis starting at 7–13 weeks post‐placement (WPP) and processed using the McMaster method to calculate ascarid egg shedding. Flocks were treated after the threshold of 200 eggs per gram of faeces (EPG) was reached.

**Results:**

The highest initial faecal egg count was 6700 EPG at 11 WPP, whereas the lowest was 50 at 8 WPP. The longest delay to detect *A. galli* was 7 weeks. The lowest and the highest number of treatments were four and six, respectively. The shortest and longest periods between any two treatments were 5 and 22 weeks, respectively.

**Conclusions:**

These results suggest that monitoring for *A. galli* should start at approximately 7 WPP and should be repeated every 8 weeks until hens are 50 weeks old. Treatment should be given only if moderate to high faecal egg counts are observed. Treatments after this point may be repeated every 8 weeks without eventually performing a faecal test. These findings provide practical support to veterinarians and egg producers dealing with ascarid worm infection in laying hens in their production stage.

## INTRODUCTION

Within the past decade, increasing attention has been drawn to roundworm infections in laying hens. The reason for this is the escalating occurrence of *Ascaridia galli* and *Heterakis gallinarum* as a result of the ban on traditional cage systems according to the changed regulations in the EU (Directive 1999/74/EC).^1^, ^2^, ^3^, ^4^ Due to the nature of the alternative housing systems (i.e., floor and enriched cage systems), hens are in increased contact with their own faeces, which can contain parasite eggs that are excreted into the environment. It has been shown that ascarid eggs can survive in poultry barns for an extended period of time provided that optimal conditions are met.^5^ The high fecundity of female *A. galli* worms together with the build‐up of infection pressure in flocks can lead to persistent problems unless infections are properly controlled.^6^

Currently, only benzimidazoles such as flubendazole and fenbendazole (FBZ) are available in the EU for the treatment of roundworms in laying hens. A primary concern about the use of anthelmintics is always the risk of the development of resistance to these compounds. In ruminants and horses, this has already become a major problem requiring ongoing investigations into the underlying molecular mechanisms of resistance.^7^, ^8^ While the evidence presented thus far suggests a lack of anthelmintic resistance (AR) in *A. galli*, it has been reported in *Ascaridia dissimilis* and *H. gallinarum*, two closely related parasites to *A. galli*.^9^, ^10^, ^11^

Many years ago, two concepts were launched to promote the long‐term sustainable use of deworming agents: (i) targeted treatment (TT), where the whole group is treated based on knowledge of the extent of the infection, and (ii) targeted selective treatment, where individual animals within the grazing group are treated, which of course requires the correct identification of the animals that need treatment.^12^, ^13^ These approaches are used to prevent worm‐related adverse production effects while preserving the long‐term efficacy of deworming agents by maintaining a pool of parasites in refugia (i.e., parasites that are not exposed to the drug).^14^ For practical reasons, a typical strategy used by producers for controlling *A. galli* involves the treatment of all birds in a flock simultaneously. Moreover, since only benzimidazoles are approved for use against roundworms in chickens in the EU member states, rotation or combination of different drug classes is not possible.

Surprisingly, few studies have addressed the importance of the previously mentioned worm control programmes for poultry.^15^, ^16^ The authors previously reported on the application of a TT strategy to control *A. galli*, which resulted in lower worm burden and individual and cumulative parasite eggs per gram of faeces (EPG) levels compared with control groups.^16^ We used an arbitrary threshold of 200 EPG as an indication for treatment. The concept was based on early detection of roundworm eggs and repeated treatments with anthelmintics if necessary to prevent the build‐up of infection in chicken flocks. Moreover, hens in the TT group had better egg production, feed conversion ratio and plumage condition.^17^ For this approach to be adopted, more studies are needed on multiple commercial farms.

Current recommendations on how to use anthelmintics to control *A. galli* on Swedish farms include the following: a faecal sample should be taken at 35–40 weeks of age, followed by an initial treatment if positive; the second treatment should be done 6–8 weeks after the first and thereafter the treatment should be repeated every 8 weeks; producers are also urged to thoroughly clean and disinfect the empty barns before the start of the next production round, even though there are no uniform guidelines yet on how this should be done (Swedish Egg Association, Marike Gunnarsson, personal communication). Some concerns about the current recommendations are that (i) late initiation of monitoring can potentially lead to accumulation of parasite eggs in the environment, causing increased infection pressure in the flock, and (ii) treatment based on time schedules increases the risk of anthelmintic use in flocks where the infection level is still negligible.

This observational study, therefore, set out to assess the efficacy of the TT strategy on several commercial laying hen flocks in Sweden by investigating treatment frequencies and parasite egg output during the full production cycles; we also aimed to propose a new deworming routine based on the obtained data and the existing knowledge on infection dynamics of *A. galli*.

## MATERIALS AND METHODS

### Study flocks

The study was carried out between 2019 and 2021 in eight flocks on five commercial farms that were recruited with the help of the Swedish Egg Association (www.svenskaagg.se). For the flocks to be included in this study, they had to meet the criteria of (1) having at least 1000 birds, (2) having a history of *A. galli* and (3) having flocks recently placed (early production cycle). Information about the included flocks is presented in Table 1. Feed and water were supplied ad libitum during the entire production cycle.

**TABLE 1:** Overview of the included flocks in the study

Flock	Farm	No. of birds/flock	Age at placement (weeks)	First sample (WPP)	Production system	Project timeframes
a	F1	9281	17	11	Free range	30 JUL. 2019–11 MAY 2020
b	F1	8200	15	13	Free range	30 JUL. 2019–11 MAY 2020
c	F1	17,400	16	11	Indoor	28 JUL. 2019–1 AUG. 2020
d	F1	27,800	17	11	Indoor	28 JUL. 2019–1 AUG. 2020
e	F2	37,200	17	11	Indoor	20 SEP. 2019–14 AUG. 2020
f	F3	13,546	15	8	Indoor	20 AUG. 2019–2 NOV. 2020
g	F4	18,140	17	7	Indoor	22 OCT. 2019–28 DEC. 2020
h	F5	1342	15	7	Indoor	10 DEC. 2019–16 MAR. 2021

*Note*: All of the birds were housed in an aviary system and were all Bovans Robust hybrid.

Abbreviation: WPP, weeks post‐placement.

### Sampling and coprological analysis

No birds were directly handled during this study, and samples were collected by trained staff to reduce the introduction of unnecessary stress. To assess the overall barn contamination level with *A. galli* eggs, biweekly pooled faecal samples from the litter belts were obtained. Faecal samples (pooled samples ≈800 g each) were collected on four plastic trays (20 × 27 cm) placed on litter belts under the slats. Trays were evenly distributed in each barn so that the collected faeces would be representative of the whole flock. The farmers were asked to run the belts a day before they placed the trays. Upon receiving the samples, the contents of the trays were thoroughly mixed, and two subsamples (3 g of faeces each) from each tray (i.e., a total of eight subsamples from the four trays) were analysed with the McMaster method to calculate ascarid egg shedding (EPG) using an analytic sensitivity of 50 EPG. Parasite egg enumeration was performed under a microscope at 10× magnification.

### Anthelminthic treatments

The results of the McMaster analyses were communicated to the producers. Treatment was performed when the biweekly EPG values surpassed the preassigned threshold of 200 EPG. Hens were then dewormed by the producers with fenbendazole (Panacur AquaSol, Intervet AB, Sweden, 1 mg/kg bodyweight), which was administered in drinking water for 5 consecutive days according to the manufacturer's recommendation. The farmers were provided with instructions on how to perform drug administration.

### Statistical analysis

Data were compiled in Microsoft Excel (Microsoft Corporation, Redmond, WA, USA) and then incorporated and analysed using JMP Pro version 16 (SAS Institute Inc., Cary, NC, USA) and GraphPad Prism version 8 (GraphPad Software Inc., La Jolla, CA, USA) to build graphical illustrations. The production period was divided into three phases (P1–P3) based on the production performance (%lay) (www.bovans.com/documents/1394/Bovans_White_CS_product_guide_alternative). P1: point of lay until 36 weeks (≥96%), P2: 37–56 weeks (90%–95%) and P3: 57 weeks until end of lay (≤90%).

Data were first transformed in GraphPad Prism version 8 using the normalise function. Since the number of observations was not equal in each flock, these data were analysed by fitting a mixed model. Tukey's Honest Significant Difference (HSD) all pairwise comparisons were used to determine whether differences between flocks’ mean EPG in different production stages were significant. The significance and confidence levels were 0.05 and 95% confidence intervals, respectively.

## RESULTS

**FIGURE 1:**
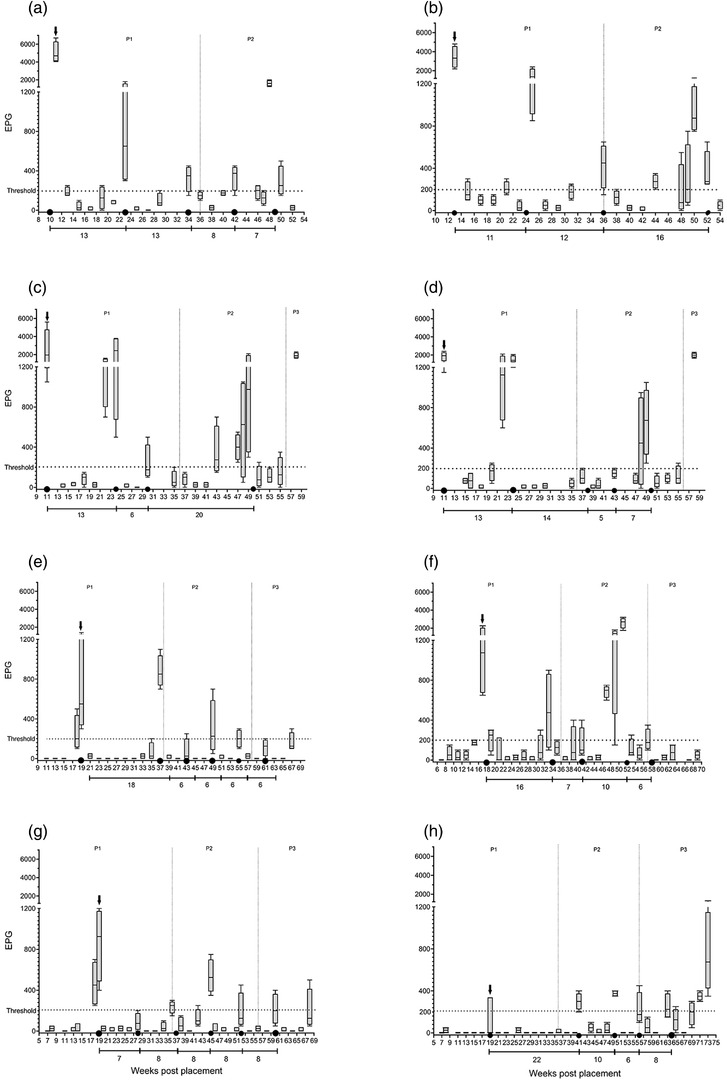
Median egg per gram faeces (EPG) obtained from a biweekly faecal analysis in eight laying hen flocks (a–h). Black circles indicate treatment occasions. Black arrows point to the highest EPG level before the first treatment. Horizontal dashed lines mark the 200 EPG threshold. Vertical dashed lines separate the three production periods (P1–P3). The numbers underneath the segmented lines show the time intervals between any two treatment occasions

The results of the McMaster analysis (expressed as the median EPG for the eight subsamples per sampling occasion) and treatment occasions for each flock are presented in Figure 1a–h. The highest initial EPG counts were observed in flocks a (6700) and c (5600) at 11 weeks post‐placement (WPP). The lowest initial EPG count was obtained in flocks g (50) and h (50) at 8 WPP. The longest interval to detect *A. galli* was seen in flock e, where parasite eggs were first detected at 18 WPP. The lowest number of treatments was achieved in flocks b and c with four treatment occasions, while the highest was achieved in flocks e and g with six treatment occasions. The shortest period between any two treatment occasions was in flock d (5 weeks), whereas the longest was observed in flock h (22 weeks).

After each treatment, it was noted that the increase in the EPG values was not gradual; instead, sharp increases in faecal egg count (FEC) were detected after a period of relatively low EPG measurements. With some exceptions, most samples were positive for *A. galli* 2 weeks after the end of the treatments. After the first recorded EPG value that surpassed the threshold (marked with black arrows in Figure 1), which was followed by the first treatment, the rest of the observed EPG values stayed below that initial level except for flocks f and h, in which some EPG values (measured 51 WPP and 41 WPP onwards) exceeded the EPG level before the first treatment.

**FIGURE 2:**
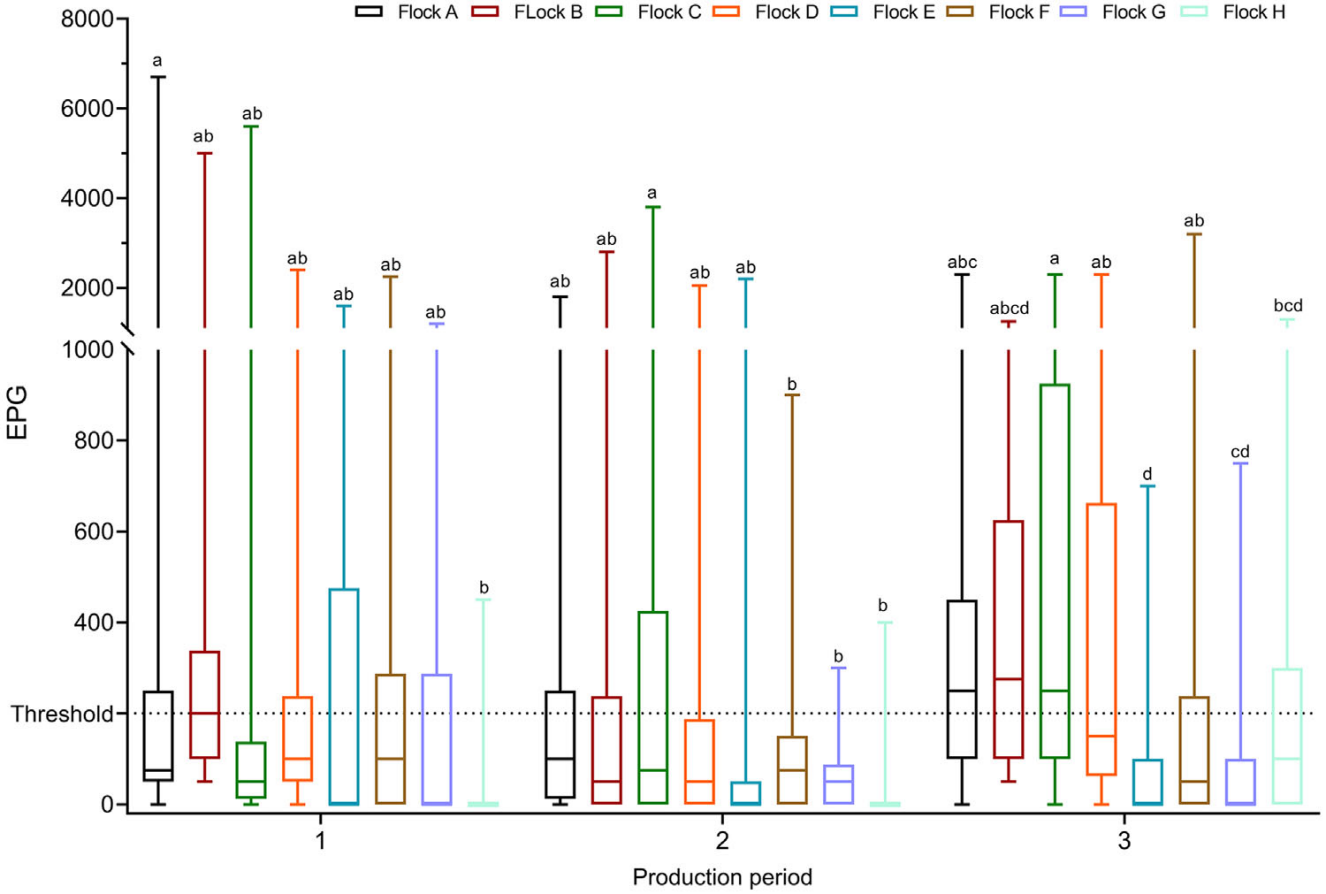
Median egg per gram faeces (EPG) calculated from all the samples from each laying hen flock for every production period. Flocks with the same letter are not significantly different (Tukey HSD All Pairwise Comparisons). The horizontal dashed line marks the 200 EPG threshold

The median EPG for each production period for each flock and the Tukey HSD. All pairwise comparisons are shown in Figure 2. The median values for most flocks were below our assigned threshold of 200 EPG except for P3, where the median values for flocks a, b and c were slightly above the threshold. Generally, distributions were positively skewed because the whiskers and half‐boxes were longer on the top side of the median than on the bottom side. The most concentrated distributions were observed in P2.

## DISCUSSION

In this observational study, the effect of early detection and timely treatment of the roundworm *A. galli* on the overall infection level in several laying hen flocks was measured. Our results show that worm infection can be established in a flock as early as 7–10 WPP with EPG values as high as nearly 7000 EPG (flock a). This highlights the importance of early detection of the infection and treatment if necessary. Moreover, no parasite eggs were detected until 18 WPP in flock e, rendering treatments on fixed time intervals inefficient. The number of treatment occasions varied from four to six times in our study, indicating that treatment can be tailored to meet individual flock requirements for worm control.

The high level of biosecurity practiced on rearing farms in Sweden reduces the risk of the introduction of *A. galli* with pullets to the production sites. This is in accordance with the results of the Swedish national control programme for roundworms (unpublished data, National Veterinary Institute, SVA, Sweden). Thus, it is unlikely that parasite infection was introduced to the farm by infected pullets. On the other hand, it is plausible to assume that hens become infected by residual infective eggs in the barn. In addition, hens in flocks a and b had access to outdoor runs, providing a potential reservoir for the infection. This could explain the very high initial EPG level observed in these two flocks already at 11 WPP. It has been shown that outdoor runs can act as an important infection source for *A. galli*.^18^, ^19^, ^20^ For flocks c and d (with no access to the outdoors), since they were on the same farm as flocks a and b, we suggest that such high EPG levels at 11 WPP might be a result of unsuccessful barn cleaning or cross‐contamination between flocks due to suboptimal biosecurity at the farm level. Our intention was to start the sampling at 7 WPP in all flocks, but due to logistical reasons, the first sampling was delayed in some flocks (a, b, c and d). If the infection is not detected and managed properly, such high environmental contamination with parasite eggs early on during production can negatively affect the host's welfare and productivity.^17^, ^21^ The hormonal and immune status of the hens, related to laying activity, seems to have a significant negative impact on resistance against worms.^22^ This further highlights the importance of early detection of the infection. Since the prepatent period for *A. galli* is between 4 and 6 weeks, it is valuable if the first parasitological faecal analysis is performed at 6–7 WPP.^23^ Delaying the first faecal analysis can result in a significant increase in the infection pressure in poultry barns.^16^ Our observation of early establishment of the infection agrees with previous observations of the *A. galli* infection pattern in Swedish laying hen flocks.^15^, ^16^

Late onset of infection has also been reported in connection with effective cleaning and disinfecting of the barn before placement of the next flock.^15^ However, it is important to note that a negative McMaster result does not mean a lack of infection but rather indicates that the number of parasite eggs is less than 50 in a gram of faeces. Negative FEC may also be observed when adult worms in the intestines are all either males or females. Since we analysed pooled faecal samples (collected from the entire barn), delayed onset infection in our study is possibly a result of a low infection level below the detection limit of our test rather than hens harbouring exclusively male or female worms. Moreover, EPG counts in flocks g and h stayed below our threshold until 18 and 19 WPP, respectively. Together, these results clearly show that frequent monitoring and faecal analysis can reduce the number of required treatments as opposed to fixed treatment intervals.

The McMaster technique has been used extensively for many years in parasitology for quantitative faecal analysis. It can be modified by the amount of faeces and the volume of flotation solution to obtain different detection levels, of which 5, 20, 50 and 100 are the most employed. The FLOTAC and the Mini‐FLOTAC techniques were developed with the aim of combining sensitivity and low cost to allow good diagnostics in resource‐limited laboratories, and it has been shown that both techniques have more sensitivity than McMaster.^24^, ^25^, ^26^, ^27^ It is unfortunate that this study did not include the aforementioned diagnostic methods because they were not available in our laboratory at the time of this study. A natural progression of this work is to compare different diagnostic methods that can be utilised for the assessment of roundworm infection in poultry.

All flocks were positive for *A. galli* 2 weeks after treatment on repeated occasions. The treatment efficacies measured roughly from FEC reduction ranged between 53% and 100% (data not shown). It is still unclear whether these positive samples were the result of a suboptimal sampling procedure, suboptimal efficacy of FBZ given in drinking water or noninfective/nonviable passant eggs or a combination of the three. As described earlier, sampling was performed in a systematic way in all flocks. However, to the best of our knowledge, no parasitological study has touched upon sampling routines in poultry barns. Both conventional (McMaster or flotation) and molecular parasitological diagnostic tools rely on samples that are representative and are of high quality.^28^ Practical and correct sampling procedures remain one of the greatest challenges of any parasite control strategy and undoubtedly need further investigation in chickens. Regarding treatment efficacy, it has been shown that the efficacy of FBZ against *A. galli* was lower when the drug was administered to hens through drinking water (81%) than when offered individually (99%).^29^ It should be appreciated that our estimation was based on pooled faecal samples and obtained under field conditions.

Awareness of passant ascarid eggs is not recent and has been described previously as false‐positive egg counts.^23^, ^30^, ^31^ In the present study, we observed 50–200 EPG on several occasions 2 weeks after the treatment, which is more in line with our previous report.^23^ However, a much larger range (20–1060 EPG) for false‐positive egg count for the porcine ascarid *Ascaris suum* has been reported.^30^ Accounting for passant eggs is an important element in setting a threshold for anthelmintic treatment. Information on the choice of a cut‐off value used by veterinarian surgeons and parasitologists for the treatment of *A. galli* is scarce. Moreover, a much‐debated question is whether *A. galli* egg excretion, quantified as EPG, is correlated with the actual worm burden. A positive correlation has been shown for *H. gallinarum*.^32^ This also needs further investigation in *A. galli*. Except for a few occasions, reductions in the EPG values after each treatment were generally significant. This indicates that treatment in most cases effectively lowered the parasite egg output. However, we also detected rather sharp increases in FEC after a period of relatively low EPG (due to treatment). This was more prominent in flocks a, b, c, d and f, wherein various cases of up to a 1500% increase in FEC were observed within a 2 weeks period. Whether this sudden increase is part of the natural course of the infection or perhaps a result of the suboptimal sampling procedure, this underestimated or overestimated EPG can significantly affect the number of treatments. As discussed above, this is particularly important and needs further detailed investigation.

Except for a few sampling occasions (in flocks f and h), none of the EPG values surpassed the initial peak observed between 10 and 19 WPP. In the case of flock h, the owner chose not to treat the flock towards the end of the laying cycle. This indicates that if an infection is detected early, with a proper treatment routine, the level of contamination for the entire production cycle can be potentially limited to the level observed at the beginning of the cycle. While we did not measure worm burdens in the current study, it has been reported that there is no correlation between hen stocking rate and *A. galli* worm burden at the endpoint of an experiment.^33^ However, that study reported that the group with the high stocking rate had a higher EPG output until day 200 postinoculation (PI) compared to the low and medium stocking rate groups.^33^ If we presume that pullets become infected upon arrival, then the 200 days PI roughly translates to 45 weeks of age. It seems therefore reasonable to suggest monitoring the flocks every 7–8 weeks until hens are approximately 45 weeks old and treat only if necessary, irrespective of the flock size and density.

Due to logistical constraints, some treatments were delayed. For example, in flocks b and c, the fourth treatment, and in flock f, the third treatment was delayed for several weeks. This resulted in an exponential increase in the EPG levels, which did not seem to be age related. This shows that timing is crucial when treating worm infections. Due to its high fecundity, *A. galli* can contaminate the environment with massive numbers of eggs (∼40,000 per day per worm) every day, and the treatment is delayed.^6^ This also agrees with our earlier observations, which showed that the EPG level in untreated control flocks increased rapidly when measured at biweekly intervals.^16^ However, the findings of the current study do not support a previous report suggesting that resistance to *A. galli* in older chickens may be expressed as reduced worm fecundity.^34^ This inconsistency may be because pooled faecal samples (reflecting the flock EPG levels) were used in our study as opposed to individual faecal samples. The time between treatment occasions also varied somewhat between the different flocks. Although no general pattern was observed, it seemed that the treatment intervals towards the end of the production cycle became shorter compared to the beginning of the cycle. The reason for this may be that parasite eggs accumulate towards the end of the production period, causing the infection to be more widespread in the flock than in earlier periods. For this reason, it is safe to say that treatment after 45 weeks of age can be repeated every 8 weeks without faecal analysis, provided that the earlier faecal analysis resulted in moderate or high EPG counts.

Evidence of AR in the closely related nematodes *A. dissimilis* and *H. gallinarum* has been described in turkeys and chickens (broilers) in the USA.^10^, ^11^ A reasonable approach to tackle this issue could be to monitor resistance development in *A. galli*. Evaluation of the efficacy of anthelmintics on each farm is essential to any ongoing control programme. This evaluation may be done systematically under field or laboratory conditions or performed informally by farmers themselves investigating the intestinal content of a few birds posttreatment.^35^ It is, however, important to be aware that culled sick birds may not be suitable for efficacy assessments, as their antemortem condition leading to their death could potentially affect intestinal worm establishment and therefore lead to an incorrect estimation of anthelmintic efficacy.

Even though TT potentially promotes repeated treatment, the authors would like to highlight that anthelmintics should be used only when necessary and that treatment should be justified based on parasitological data. To achieve sustainable results, anthelmintic use should be combined with improved management, such as barn hygiene, strict biosecurity and, most importantly, correct dosing of hens. Finally, the suggested 200 EPG threshold and the treatment interval of 8 weeks, which is greater than the prepatent period of *A. galli*, should theoretically help maintain the parasite population in refugia, which in turn prolongs the efficacy of the current anthelmintics in the market. Although this study focuses on enhancing the current guidelines for the control of *A. galli* infection in laying hens in Sweden, the insights gained from this study may well be of help to egg producers in other countries. These findings suggest several courses of action for enhancement of the control of *A. galli*. We recommend that parasitological investigation should start as early as 7 WPP (approximately 22 weeks of age in Sweden) in the form of FEC analysis. Until the hens are 45–50 weeks old, faecal analysis should be repeated at 7–8 weeks intervals; treatment should be considered only if FEC shows moderate or high EPG. In this way, unnecessary use of anthelmintics will be avoided, and treatment will be tailored for each flock. Timing is essential; therefore, communicating the results to the producers is important. The treatment can thereafter be performed every 8 weeks without faecal analysis if evidence of an established infection is provided. These recommendations are intended for laying hens in their production stage since younger birds at the growing stage were not assessed in this study. If possible, producers should regularly monitor for *A. galli* throughout the production cycle. If not, we recommend that samples should be taken towards the end of the production cycle to assess the infection pressure in the barn. Such information will aid decision making on the type of cleaning practices required when the barn is empty.

## CONFLICTS OF INTEREST

The authors declare they have no conflicts of interest.

## AUTHOR CONTRIBUTIONS

All authors contributed to the planning, execution and writing of this manuscript.

## ETHICS STATEMENT

No ethical review was required given that faecal samples were analysed as part of routine monitoring.

## Data Availability

The data that support the findings of this study are available on request from the corresponding author.
